# Impact of the warm summer 2015 on emergency hospital admissions in Switzerland

**DOI:** 10.1186/s12940-019-0507-1

**Published:** 2019-08-15

**Authors:** Martina S. Ragettli, Ana M. Vicedo-Cabrera, Benjamin Flückiger, Martin Röösli

**Affiliations:** 1 Swiss Tropical and Public Health Institute, Socinstrasse 57, P.O. Box, CH-4002 Basel, Switzerland; 2 University of Basel, Basel, Switzerland; 3 Department of Public Health, Environments and Society, London School of Hygiene & Tropical Medicine, London, UK

**Keywords:** Heat, Heatwave, Hospitalization, Morbidity, Temperature

## Abstract

**Background:**

Only a few studies have examined the impact of a particular heat event on morbidity. The aim of this study was to evaluate the impact of the warm summer 2015 on emergency hospital admissions (EHA) in Switzerland. The summer 2015 ranks as the second hottest after 2003 in the history of temperature observation in Switzerland.

**Methods:**

Daily counts of EHA for various disease categories during summer 2015 were analyzed in relation to previous summers in Switzerland. Excess EHA for non-external causes during summer 2015 (June–August) were estimated by age group, gender, geographic region and disease category by comparing observed and expected cases. The latter were predicted from strata-specific quasi-Poisson regression models fitted to the daily counts of EHA for years 2012–2014.

**Results:**

Over the three summer months in 2015, an estimated 2.4% (95% confidence interval [CI] 1.6–3.2%) increase in EHA (non-external causes) occurred corresponding to 2,768 excess cases. Highest excess EHA estimates were found in the warmest regions (Ticino [8.4%, 95% CI 5.1–11.7%] and the Lake Geneva region [4.8%, 95% CI 3.0–6.7%]) and among the elderly population aged ≥75 years (5.1%, 95% CI 3.7–6.5%). Increased EHA during days with most extreme temperatures were observed for influenza and pneumonia, certain infectious diseases and diseases of the genitourinary system.

**Conclusions:**

Summer 2015 had a considerable impact on EHA in Switzerland. The daily number of EHA mainly increased due to diseases not commonly linked to heat-related mortality. No excess morbidity was found for cardiovascular and most respiratory diseases. This suggests that current public health interventions should be reevaluated to prevent both heat-related illness and deaths.

**Electronic supplementary material:**

The online version of this article (10.1186/s12940-019-0507-1) contains supplementary material, which is available to authorized users.

## Introduction

Heatwaves are widely recognized as an important public health concern. Several studies reported large excess mortality attributed to heatwaves occurring in different locations across the globe since the beginning of the century [1–5]. The elderly, people with chronic diseases and low socioeconomic status are considered the most vulnerable population groups to adverse health effects of exposure to high ambient temperatures [6, 7]. The ability of elderly populations to cope with heat is affected by a limited capacity of thermoregulation, pre-existing diseases and medication. Main causes of heat-related deaths include cardiovascular, cerebrovascular and respiratory diseases [8–10]. Understanding the impact of heatwaves on human health and the effect of adaptation strategies is gaining importance in view of climate change, progressing urbanization and the aging of the population [11]. It is very likely that the frequency, duration and intensity of heatwaves is increasing in the following decades, affecting urban populations more than others [12].

Heatwaves are not only related to excess mortality, but also with increased morbidity risk. Time series studies have found positive associations between heat and emergency hospital admission (EHA) for various heat-related conditions (e.g. heat stroke, heat exhaustion and dehydration) and other diseases not specifically coded as heat-related such as respiratory diseases and renal causes [7, 13–20]. The magnitude of effect for various diseases varies by age structure of the population, local climate, available health infrastructure and adaptation strategies [13, 21].

Only a few studies evaluated the impact of a particular heat event on morbidity [10, 13]. Reported impacts on EHA are generally lower than those for mortality [3]. For example, the 2003 heatwave in England was linked with a 1% increase in all-cause EHA and a 17% increase in mortality compared to the same time period in the previous 5 years [22]. During a heatwave in New South Wales, Australia, in 2011, all-cause EHA and mortality increased by 2 and 13%, respectively [23]. Although lower in relative terms, absolute numbers of excess emergency visits can be large. In California, an 18-day heatwave in 2006 was associated with 16,166 excess emergency department visits with substantial increases for renal causes, diabetes and electrolyte imbalance. In this study by Knowlton et al. [24] the excess cases refer to the difference in the numbers between heatwave days and a near-term summer reference period of the same duration in the same year. Furthermore, comparisons of temperature thresholds for different health events revealed lower triggering points for heat-related emergency department visits than heat-related mortality [25].

Europe was hit by an extreme hot summer in 2015. Central and Eastern Europe were particularly affected. In Switzerland, summer 2015 ranks as the second warmest since the beginning of temperature observation in 1864. It was characterized by three major heatwaves, two in July (July 1–7, July 16–24) and one in August (August 5–9). During these heatwaves, mean maximum daytime temperatures (Tmax) between 33 °C and 36 °C were measured in the lowlands and reached the record temperature of 39.7 °C in Geneva on July 7. The month of July was, in many parts of the country, the hottest month in the history of temperature observation. Overall, the mean temperature during June to August was 2.4 °C warmer compared to the reference period 1981–2010. Higher mean summer temperatures were only recorded during the hot summer 2003, being around 1 °C above the value measured in summer 2015 [26]. Both summers had a significant impact on mortality in Switzerland. Excess mortality of 6.9 and 5.4% were respectively estimated in 2003 [27] and 2015 [2]. After the heatwave in 2003, the public awareness of heat threats was improved in Switzerland. Many cantons (Swiss term for *counties*) have implemented public health measures to prevent adverse heat-related health impacts. However, existing public health strategies are mainly oriented on heat-related mortality. The effect on morbidity and EHA in Switzerland remains unclear. Summer 2015 is an opportunity to evaluate the effect on morbidity in a central European country with a temperate climate. An understanding of how heatwaves affect short-term morbidity may help to improve adaptation strategies to prevent both heat-related excess morbidity and mortality.

The aim of this study was to evaluate the impact of the warm summer 2015 on morbidity in Switzerland. In particular, we assessed whether the high summer temperatures had a measurable impact on specific disease categories and population subgroups by analyzing EHA in all Swiss hospitals between June and August 2015 and comparing them to previous years.

## Methods

### Study description

First, daily counts of EHA in the total population and for different age groups and various disease categories during the warm season (May to September) 2015 were descriptively compared to the same time period of the ten previous years (2005–2014). Disease categories (Table 1) were selected for which associations with heat are plausible according to previous heatwave studies on morbidity [13, 25].

**Table 1:** Number of emergency hospital admissions during the summer months for selected diagnoses in Switzerland during the hot summer 2015

Diagnose	ICD-10-Code	June–August 2015					
		Total	Per 100,000 inhabitants^a^	Daily admissions			
				Mean	SD	Min	Max
Total, all-cause	A00-Z99	144′687	1′739	1′573	180	1′197	1′927
Total, non-external causes^b^	A00-R99, T67	117′280	1′410	1′275	169	965	1′581
Certain infectious and parasitic diseases	A00-B99	8′148	98	89	14	54	128
Mental and behavioral disorders	F00-F99	11′362	137	124	23	65	160
Diseases of the nervous system	G00-G99	4′295	52	47	9	24	76
Diseases of the circulatory system	I00-I99	17′293	208	188	31	110	252
Ischemic heart diseases	I20-I25	3′850	46	42	9	16	64
Acute myocardial infarction	I21	2′814	34	31	7	14	48
Cerebrovascular diseases	I60-I69	3′355	40	36	8	20	53
Diseases of arteries, arterioles and capillaries	I70-I79	938	11	10	5	2	22
Diseases of the respiratory system	J00-J99	8′399	101	91	18	58	138
Upper respiratory tract	J00-J06, J30-J39	1′248	15	14	3	6	20
Influenza and pneumonia	J09-J18	3′284	39	36	9	16	55
Lower respiratory tract	J20-J22, J40-J47	2′537	30	28	9	12	56
Diseases of the digestive system	K00-K93	16′159	194	176	29	114	236
Diseases of the genitourinary system	N00-N99	8′758	105	95	16	64	131
External causes of morbidity ^c^	S00-T98, V01-Y84, Z00-Z99, U00-U99	27′474	330	299	32	210	374
Effects of heat and light	T67	67	0.8	0.7	1.4	0	9

^a^Population on December 31

^b^External causes of morbidity are excluded expect effects of heat and light (T67). Includes diseases not specifically assessed in this study: Neoplasms (C00-D48); Diseases of the blood and blood-forming organs and certain disorders involving the immune mechanism (D50-D90); Endocrine, nutritional and metabolic diseases (E00-E90); Diseases of the eye and adnexa (H00-H59), Diseases of the ear and mastoid process (H60-H95); Diseases of the skin and subcutaneous tissue (L00-L99); Diseases of the musculoskeletal system and connective tissue (M00-M99); Pregnancy, childbirth and the puerperium (O00-O99); Certain conditions originating in the perinatal period (P00-P96); Congenital malformations, deformations and chromosomal abnormalities (Q00-Q99); R00-R99 (Symptoms, signs and abnormal clinical and laboratory findings, not elsewhere classified)

^c^Includes the following ICD-10 codes: Injury, poisoning and certain other consequences of external causes (S00-T98; External causes of morbidity (V01-Y84); Factors influencing health status and contact with health services (Z00-Z99); Codes for special purposes (U00-U99)

*SD* standard deviation, *Min* minimum, *Max* maximum

Second, excess morbidity during summer 2015 was assessed. The number of excess EHA by sex, age group, geographic area and disease categories were estimated by comparing the observed and expected cases (i.e., the number of EHA that would have been expected during a normal summer without heatwave) between June and August. The expected cases were predicted using data of the three previous years (2012–2014). A recent comparison period was chosen to predict the excess morbidity during summer 2015 to exclude other factors related to the number of EHA that are difficult to control for in a dynamic health system (e.g. changing reimbursement rules for cost control).

### Health and population data

The anonymized individual EHA from 2005 to 2015 were provided by the Federal statistical office of Switzerland (Medical Statistics of Hospitals). All cases among Swiss residents were aggregated into daily counts by age group (0–14, 15–64, 65–74, ≥75 years), sex, specific disease categories according to the International Classification of Diseases (ICD, revision 10) and geographic area. The disease categories and corresponding ICD-10 codes are listed in Table 1. The geographic areas were based on the seven main regions in Switzerland: Northwestern Switzerland, Espace Mittelland (i.e. Swiss plateau), Lake Geneva, Zurich, Ticino, Central Switzerland and Eastern Switzerland (Table 2). These regions represent similar environmental and population characteristics. Similar to Petitti et al. [25], our analyses based on total non-external EHA (A00-R99, T67) excludes most external causes of morbidity (S00-T98, U00–99, V01-Y84, Z00–99); effects of heat and light (T67) are included as these are heat-related (Table 1).

**Table 2:** Description of the population and daily maximum daytime temperature (Tmax) and daily minimum nighttime temperature (Tmin) in the seven Swiss regions during summer months (June–August) in 2012–2014 and in 2015

Region (Cantons)	Population^a^ (%)	Station	Elevation (m)	Tmax (°C) Mean (range)		Number of days with Tmax ≥30 °C		Tmin (°C) Mean (range)		Number of days with Tmin ≥20 °C	
				2012–2014	2015	2012–2014^b^	2015	2012–2014	2015	2012–2014^b^	2015
Northwestern Switzerland (Cantons AG, BL, BS)	1 122 808 (13.6%)	Basel-Binningen	316	24.6 (12.5–37.3)	26.7 (16.6–37.0)	11	28	14.5 (5.0–20.9)	15.7 (8.4–22.3)	2	11
Espace Mittelland (Canton BE, FR, JU, NE, SO)	1 831 565 (22.1%)	Bern-Zollikofen	553	23.4 (12.0–34.1)	25.9 (16.5–36.8)	8	26	12.8 (4.0–19.6)	14.2 (6.7–20.0)	0	1
Lake Geneva (Cantons GE, VD, VS)	1 581 953 (19.1%)	Genève-Cointrin	412	25.1 (14.1–34.5)	28.1 (18.4–39.7)	11	34	14.2 (7.0–21.3)	15.7 (8.7–22.5)	1	7
Zurich (ZH)	1 456 217 (17.6%)	Zurich-Fluntern	556	23.1 (11.2–35.1)	25.8 (14.4–34.6)	7	26	14.0 (4.4–22.4)	15.6 (7.9–21.5)	2	11
Ticino (TI)	351 131 (4.2%)	Lugano	273	26.4 (16.9–33.4)	27.8 (19.3–33.9)	12	27	17.7 (10.9–24.3)	19.0 (13.1–25.4)	20	31
Central Switzerland (Cantons LU, NW, OW, SZ, UR, ZG)	786 362 (9.5%)	Luzern	454	23.8 (12.2–34.9)	26.1 (15.1–34.8)	10	26	14.2 (6.9–21.7)	15.8 (8.8–21.5)	1	7
Eastern Switzerland (Cantons AI, AR, GL, GR, SH, SG, TG)	1 148 946 (13.9%)	St. Gallen	776	20.6 (9.9–33.3)	22.8 (12.3–31.7)	1	5	13.3 (3.0–21.8)	15.1 (7.8–22.6)	3	11

^a^Total population at mid-year 2015

^b^Mean of number of days in 2012, 2013 and 2014

Cantons: *AG* Aargau, *AI* Appenzell I. Rh., *AR* Appenzell A. Rh., *BE* Bern, *BL* Basel-Landschaft, *BS* Basel-Stadt, *FR* Fribourg, *GE* Geneva, *GL* Glarus, *GR* Graubünden, *JU* Jura, *LU* Lucerne, *NE* Neuchâtel, *NW* Nidwalden, *OW* Obwalden, *SG* St. Gallen, *SH* Schaffhausen, *SO* Solothurn, *SZ* Schwyz, *TG* Thurgau, *TI* Ticino, *UR* Uri, *VD* Vaud, *VS* Valais, *ZG* Zug

Annual population data per 31st of December were obtained from the Federal statistical office. Mid-year population size for each stratum and year was estimated as the mean value of the annual population of the previous and current year.

### Temperature data

Daytime maximum (Tmax: between 5:40 am and 5:00 pm) and nighttime minimum (Tmin: between 5:40 pm and 5:40 am) temperature data, from a representative monitoring station (Swiss Monitoring Network) in an urban area in each of the seven regions, was used to describe the meteorological conditions during the summer 2015 and in previous years. A map showing the locations of the measurement stations in each region is provided in the Additional File 1: Fig. S1. The daily temperature data were collected from the IDAWEB database, a service provided by MeteoSwiss, the Swiss Federal Office of Meteorology and Climatology.

### Statistical analyses

The excess morbidity during the warm summer 2015 was computed by applying the same method that was used by Vicedo-Cabrera et al. [2] to estimate the excess mortality for the same time period in Switzerland. Briefly, quasi-Poisson models were fitted for each stratum (sex, age group, geographic area) to the daily number of EHA from 2012 to 2014. Two functions were included in the models to capture long-term and seasonal time trends in morbidity over this three-year period: a linear function of time (continuous ordered series from 1 January 2012 to 31 January 2014) and a trigonometric polynomial of sine and cosine terms (1-year period). The day of the week was also included. To account for potential trends in the population structure, the mid-year strata-specific population number was used as offset in the models. The strata-specific models were then used to extrapolate the expected number of EHA for each day from June to August 2015. Estimates of excess morbidity by sex, age group and geographic area were obtained by computing the difference between the respective observed and predicted EHA. The 95% confidence intervals (95% CI) of excess morbidity estimates were calculated by applying the delta method. According to the delta method, which is a linear approximation technique, the statistical errors of the daily numbers of expected EHA were expressed by a linear function of the statistical errors of the model parameters [28].

## Results

In all seven regions in Switzerland, mean daily Tmax and Tmin during summer 2015 was higher than the mean of the three previous summers (Table 2). All areas experienced a substantial increase in days with Tmax ≥30 °C and nights where Tmin did not drop below 20 °C. The highest summer mean Tmax (28.1 °C) and highest number of days with Tmax ≥30 °C were measured at the meteorological station in the Lake Geneva region. Also, among the seven stations, the highest daily Tmax (39.7 °C) during summer 2015 was measured in Geneva on July 7 at the end of the first heatwave. Ticino, the southern part of Switzerland, was the second warmest area (mean Tmax: 27.8 °C) and showed the highest number of nights with Tmin ≥20 °C.

**Fig. 1:**
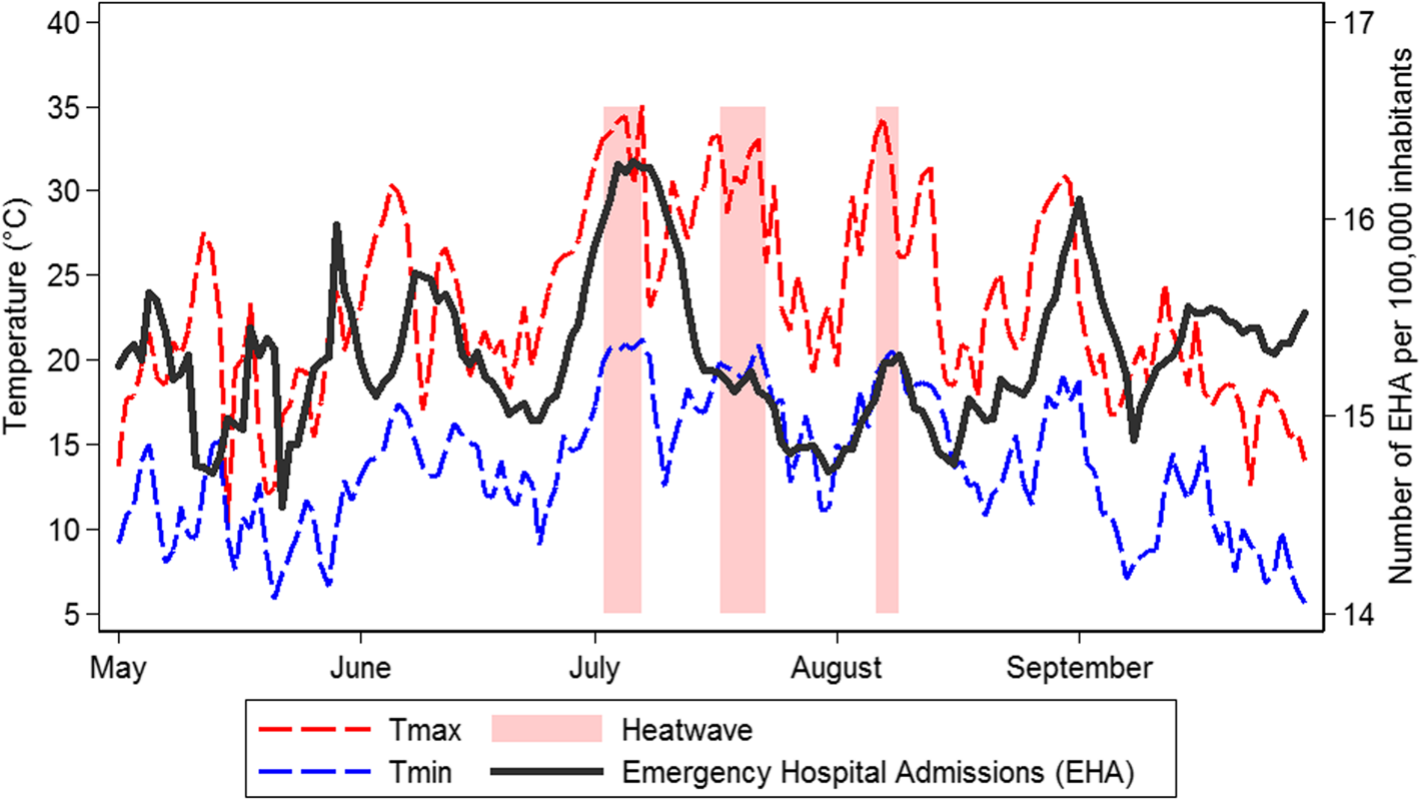
Daily number of emergency hospital admissions (EHA) per 100,000 inhabitants (7-day moving average) in Switzerland, and average maximum daytime (Tmax) and minimum nighttime (Tmin) temperature across seven regions in Switzerland during the warm season 2015

**Fig. 2:**
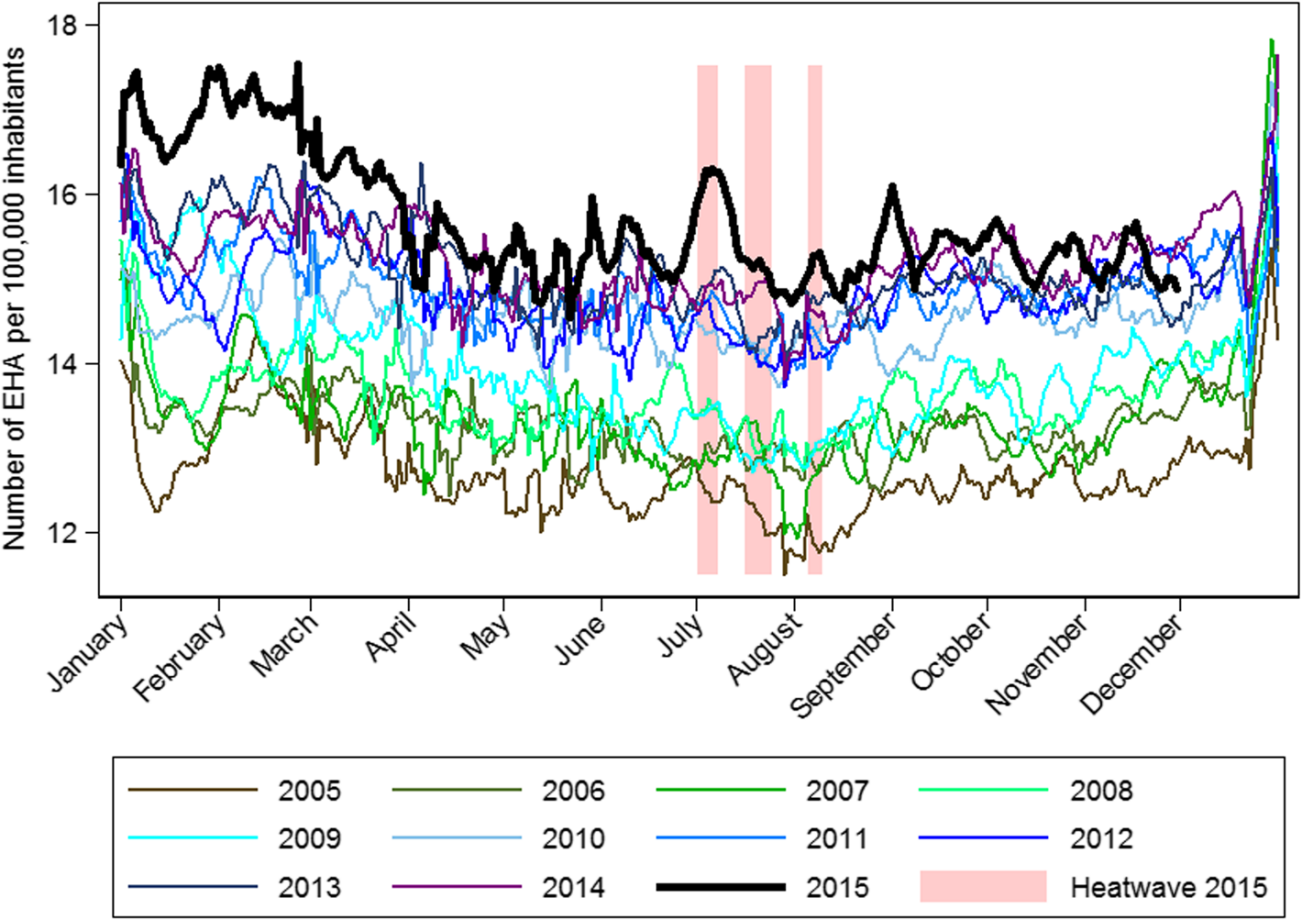
Daily number of emergency hospital admissions (EHA) per 100,000 inhabitants (7 day moving average) in Switzerland between 2005 and 2015. External causes of morbidity are excluded except effects of heat and light (ICD10-code T67)

The daily number of EHA per 100,000 inhabitants increased noticeably during the three heatwaves in Switzerland in summer 2015 (Fig. 1). The highest peak of EHA coincided with the first heatwave in July (1 July to 7 July) which was characterized by a sharp increase in both daytime and nighttime temperatures. Compared to the previous ten years, this was the most prominent peak in daily EHA counts during the warm season (Fig. 2). This was particularly the case for the oldest population (Additional file 1: Figure S2).

Table 1 describes the number of EHA by diagnosis in Switzerland during the summer months in 2015. Based on the graphical analysis of the time trends of daily EHA counts by diagnosis between 2005 and 2015 (Additional file 1: Figure S3), the peaks observed during the 2015 heatwaves derive from increases in daily rates of EHA related to influenza and pneumonia, certain infectious diseases and diseases of the genitourinary system. The most pronounced peak during the first 2015 heatwave was observed for effects of heat and light (ICD-10 T67), although these diagnoses accounted for only 0.05% of all EHA (*n* = 67). EHA related to diseases of the circulatory system decreased during the heatwaves in 2015.

**Fig. 3:**
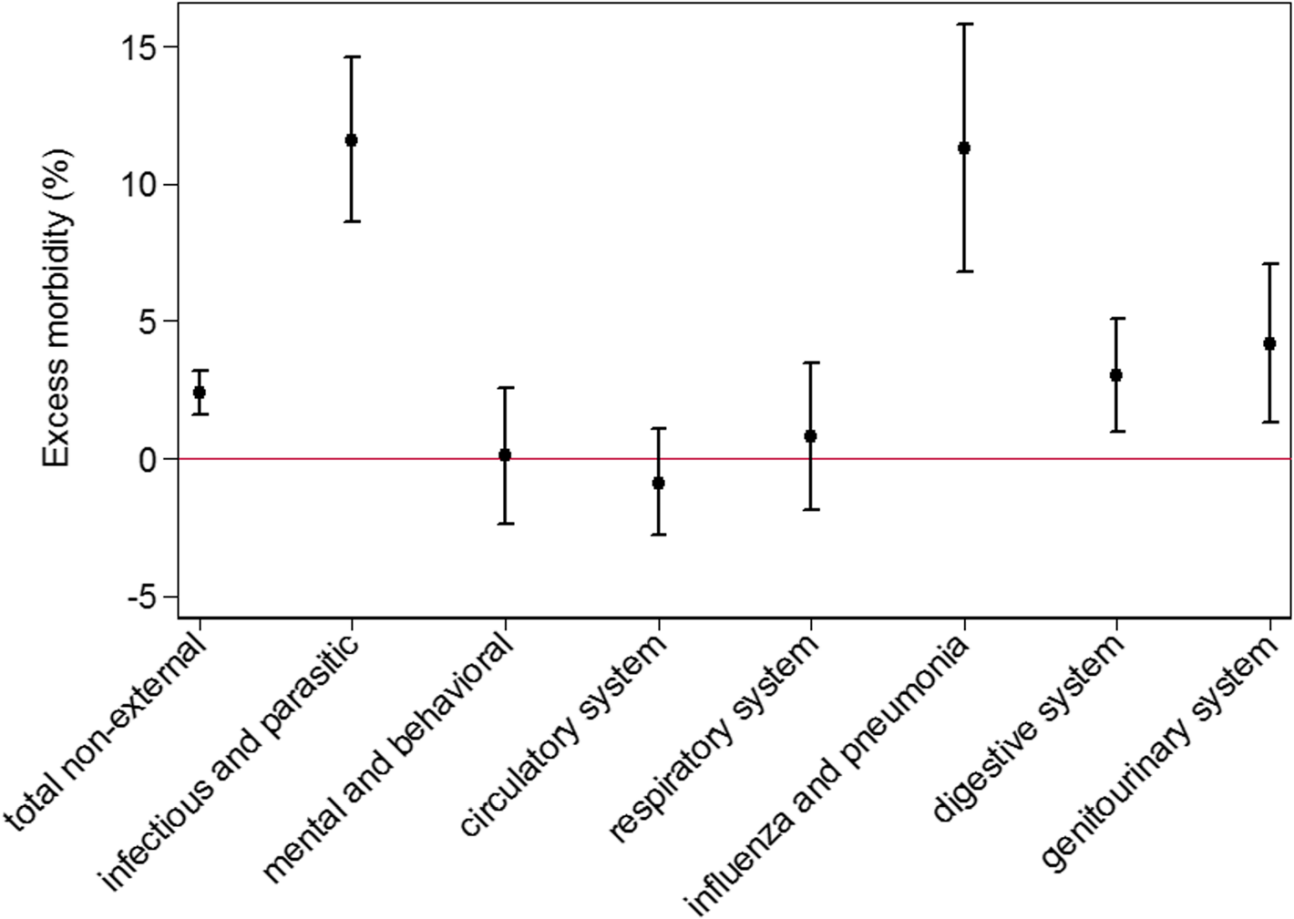
Estimated percentages of excess morbidity (with 95% confidence intervals) during summer 2015 for total non-external causes and selected diseases

A total of 2,768 (95% CI: 1,885-3,651) additional EHA were estimated during June to August 2015 considering all diagnoses without external cases of morbidity (Table 3). This corresponds to 2.4% (95% CI: 1.6–3.2%) excess morbidity compared to the three previous years accounting for trends in mortality and temporal changes in the population structure. Among the three summer months, the largest deviation was observed in July (4.0%, 95% CI: 2.9–5.1%) (Table 3). The elderly above 74 years old were most affected (5.1% during summer period and 10.2% in July), accounting for 63% of all excess cases during summer 2015. Less cases than expected were observed for children between 0 and 14 years old. No difference in excess morbidity was found between males and females, although women tend to be more affected during the hottest month July. Comparing the seven main regions, the largest deviations in EHA were estimated for Ticino (8.4%, 95% CI: 5.1–11.7%) and the Lake Geneva region (4.8%, 95% CI: 3.0–6.7%) which were also the regions with the highest measured mean Tmax. In Eastern Switzerland, the coldest region during summer 2015, no increase in EHA was observed. Excess morbidity was most evident for certain infectious diseases (11.6%, 95% CI: 8.6–14.6%), influenza and pneumonia (11.3%, 95% CI 6.8–15.8%), diseases of the genitourinary system (4.2%, 95% CI: 1.3–7.1%) and diseases of the digestive system (3.0%, 95% CI, 1.0–5.1%) (Fig. 3).

**Table 3:** Estimated excess morbidity (and 95% confidence intervals) assessed by emergency hospital admissions (EHA) in Switzerland during summer 2015 by sex, age group and region

	June–August 2015					July 2015				
	Observed (Number)	Excess (Number)	Excess (%)	95% CI	*p*-value	Observed (Number)	Excess (Number)	Excess (%)	95% CI	*p*-value
Total, non-external causes^a^	117′280	2′768	2.4	(1.6;3.2)	0.00	40′126	1′535	4.0	(2.9;5.1)	0.00
Male	53′878	1′410	2.7	(1.6;3.8)	0.00	18′119	455	2.6	(0.9;4.2)	0.00
Female	63′402	1′358	2.2	(1.1;3.2)	0.00	22′007	1′080	5.2	(3.6;6.7)	0.00
0–14 years	6′089	− 264	−4.2	(−7.3;-1.0)	0.01	1′970	− 131	−6.3	(− 11.0;-1.5)	0.01
15–64 years	58′067	1′070	1.9	(0.8;3.0)	0.00	19′502	230	1.2	(− 0.4;2.8)	0.14
65–74 years	17′455	226	1.3	(−0.7;3.3)	0.19	6′074	272	4.7	(1.8;7.6)	0.00
≥75 years	35′669	1′735	5.1	(3.7;6.5)	0.00	12′580	1′163	10.2	(8.1;12.3)	0.00
Northwestern Switzerland	17′562	38	0.2	(−1.8;2.2)	0.83	6′019	118	2.0	(−0.9;4.9)	0.17
Espace Mittelland	25′898	931	3.7	(2.1;5.4)	0.00	8′799	388	4.6	(2.2;7.0)	0.00
Lake Geneva	21′021	970	4.8	(3.0;6.7)	0.00	7′317	559	8.3	(5.6;11.0)	0.00
Zurich	20′109	302	1.5	(−0.3;3.4)	0.11	6′913	227	3.4	(0.7;6.1)	0.01
Ticino	6′496	503	8.4	(5.1;11.7)	0.00	2′260	243	12.0	(7.2;16.9)	0.00
Central Switzerland	9′649	294	3.1	(0.4;5.8)	0.02	3′260	106	3.4	(−0.6;7.3)	0.09
Eastern Switzerland	16′545	− 269	−1.6	(−3.6;0.4)	0.12	5′558	− 107	−1.9	(−4.8;1.1)	0.21

^a^External causes of morbidity are excluded expect effects of heat and light (ICD-10-code T67)

## Discussion

Our results suggest that the summer 2015 had a noticeable impact on morbidity in Switzerland. Especially in July, which was the hottest July in the history of temperature observation, strong increases (+ 4.0%) in daily rates of EHA were observed. For summer 2015, a statistically significant excess EHA rate of 2.4% was estimated (2,768 additional cases).

The highest excess morbidity estimates were found for the warmest regions in Switzerland where more severe heatwaves were registered. This suggests that a substantial part of the excess EHA is likely related to heat (although confidence intervals overlap and not all estimates are statistically significant). Several studies showed an increase in EHA for heat-related diseases on days with high temperatures in particularly warm areas, especially among the elderly [13, 14, 25]. A European study by Michelozzi et al. [29] found a higher impact of temperature on respiratory hospital admissions in the 75+ age group in Mediterranean cities compared to the cooler North-Continental cities. In other studies, however, the areas with the highest temperatures were not necessarily those with the highest excess morbidity during heatwaves. For example, during the California heatwave in 2006, Knowlton et al. [24] found that the regions with a cooler climate contributed more to the overall excess emergency department visits than expected. This might be explained by the impaired ability to cope with heat in regions with modest temperatures because of housing characteristics (building age, availability of air condition), individual behavior and impaired physiological acclimatization capacity. Such climate-related differences between regions did not seem to be pertinent in Switzerland, although the climate in Ticino and Lake Geneva region is somewhat warmer than in the other regions in Switzerland.

In addition, there are differences in the strategies to prevent heat-related mortality among cantons. Ticino and the cantons in the Lake Geneva region are amongst the most active in promoting public health measures to prevent adverse effects of hot weather and formally implemented heat-health action plans between 2004 and 2009. The analysis of excess mortality during summer 2015 by Vicedo-Cabrera et al. [2] revealed a lower estimated excess mortality for the Lake Geneva region (5.2%, 95% CI: − 0.7-11.1%) than the Swiss average (5.4%, 95% CI: 3.0–7.9%) despite substantially higher ambient temperatures. Such public health measures may have prevented heat-related mortality while their effect on EHA may be the opposite. That is, deaths might have been prevented because people were aware of the health risk and consulted the emergency department early enough. More studies are needed to determine whether policies to prevent heat-related mortality may also increase the use of health care services provided by emergency departments of hospitals.

Temperature aside, differences in health care utilization in the population among Swiss cantons may have contributed to higher increases in excess EHA in Ticino and the Lake Geneva region compared to other regions during the hot summer 2015. In fact, according to the Swiss statistics of the health care utilization, the canton of Ticino and the canton de Vaud in the Lake Geneva region reported the highest number of EHA relative to the population [30]. This may be related to differences in health infrastructure and unequally distributed socio-economic factors. As a result, people in these cantons visit the hospital emergency department more frequently than other health services such as family doctors. It is thus conceivable that in Ticino and the Lake Geneva region patients with relatively mild heat-related health problems more frequently visit an emergency department of a hospital during warm days, whereas in other cantons they consult the family doctor and do not appear in our statistics.

Consistent with results from previous literature [13, 15], the most vulnerable population group for heat-related morbidity was the elderly. A higher number of excess EHA for the age group ≥74 years compared to the younger age group was observed, in particular during the first heatwave in July. Both daytime and nighttime temperatures reached very hot temperatures. As a study on the 2003 heatwave in Paris showed, several days of high nighttime temperatures are particularly devastating for the health of the elderly population because the absence of a cool recovery time overnight [31]. In addition, as suggested in studies on heat-related mortality, the health effects of extreme temperatures are more evident in early summer due to a larger fragile population [9], a possible short-term physiological acclimatization and a behavioral adaption over the summer months [32, 33]. These factors may also explain the smaller peak on morbidity during the second and third heatwaves in July and August. The number of frail people likely decreased after the first heatwave because susceptible individuals received medical treatment, were more intensely taken care of or may have died after initial exposure to heat.

No excess morbidity due to the hot weather during summer 2015 was observed for children aged 0 to 14 years. In contrast to previous literature [20, 24], even the total non-external morbidity significantly decreased in this age group during the hottest month of July. Methodological reasons (e.g. larger age group, no cause-specific results, ecological nature of study) and preventive factors may explain the absence of excess morbidity in children during summer 2015 in this study. Recent information campaigns in child care facilities initiated by the cantonal authorities to raise awareness of potential heat-related health effects may have helped to protect the health of children. Furthermore, it cannot be excluded that the observed reduction in EHA among children is due to school holidays in July and early August.

The relative increase in EHA for summer 2015 (+ 2.4%) was somewhat lower than observed for mortality (+ 5.4%) [2]. However, the absolute numbers of additional hospital admissions (+ 2,768) was larger than the additional deaths (+ 804) estimated by Vicedo-Cabrera et al. [2]. Increased numbers of EHA during the three heatwaves were mainly found for causes that are not typically linked to heat-related mortality [8, 10]. This includes certain infectious diseases and diseases of the genitourinary system. The latter are likely related to renal morbidity. Exposure to high temperatures increases the risk for renal dysfunction resulting from dehydration and hyperthermia. Recent studies on the impact of heat on renal disease incidence have found higher risks for various renal health outcomes such as kidney stones [34], acute kidney injury and urinary tract infections [17] during hot days. For causes that are most common for heat-related mortality, however, no increased risk for EHA was identified during summer 2015. In fact, the daily number of cardiovascular EHA decreased for all investigated diagnoses during days of extreme heat. Also no clear increase in EHA related to respiratory diseases, expect for influenza and pneumonia, was observed. Increased frequencies of EHA with the diagnose ‘influenza and pneumonia’ are likely attributable to pneumonia [35] as no increased influenza incidence was reported during summer 2015 by the national authorities [36]. These observations might suggest that people suffering from most severe diseases that are often related to heat-related mortality died before they reached a hospital [14, 25, 37]. Also, it could be possible that the morbidity effects of heat, especially on cardiovascular morbidity, are smaller than those on mortality [37]. People with mild health effects may be more likely to access healthcare services other than the emergency departments of the hospitals. Further research is needed to better understand the effects of high temperatures on specific symptoms and age groups.

To the best of our knowledge, this is the first study that assessed the impact of heat on morbidity during the exceptional warm summer of 2015 that affected many European countries. A large variety of disease categories were analyzed. Unlike previous studies, we did not restrict the analyses to specific heatwave days but estimated excess morbidity for the whole summer. This ecological approach has the advantage that no definition of heatwave and no assumptions on lag effects have to be made.

Our approach to estimate excess morbidity does not assess the direct relationship between temperature and morbidity. EHA during the hot summer 2015 are compared to the number of EHA that would be expected in a normal summer without heatwaves taking into account mortality trends and changes in the population structure. Thus, an assessment of the population exposure to heat was not necessary. Most studies on the temperature-morbidity relationship used stationary data on temperature [e.g. 14, 25] which may have some limitations regarding the representativeness of the heat exposure of a given population. However, statements related to the temperature-morbidity relationship and temperature thresholds for relevant increases in morbidity risk are not possible in this study. Nonetheless, the excess EHA estimates for 2015 provide useful insights on the morbidity impact of an exceptional warm summer. Other mechanisms that may have influenced the increase in EHA such as changes in public health practice were limited by choosing a relatively short reference period. During the analyzed period (2012–2015) no significant changes in heat-health action plans were made and a general awareness of heat-related health effects in the population was assumed to be constant.

## Conclusions

This study shows that the hot summer 2015 had a significant burden on morbidity in Switzerland. Highest excess EHA estimates were observed in the warmest regions and for people aged ≥75 years. During the 2015 heatwaves, the daily number of EHA mainly increased due to diseases that are not commonly linked to heat-related mortality. This suggests that more effective public health interventions should be promoted considering both morbidity and mortality indicators to reduce the health burden of extreme heat.

## Additional file

Additional file 1: Additional tables and figures. **Figure S1.** Map of Switzerland showing the seven study regions and corresponding measurement station of temperature of the Swiss Monitoring Network. **Figure S2.** Daily number of emergency hospital admissions (EHA) (7 day moving average) in Switzerland by age group (0–14 years, 15–64 years, 65–74 years, ≥75 years) during the warm season between 2005 and 2015. External causes of morbidity are excluded except effects of heat and light (ICD10-code T67). **Figure S3.** Daily number of emergency hospital admissions (EHA) (7 day moving average) for selected diagnoses in Switzerland from 2005 to 2015. The reference years (2012–2015) for the 2015 excess morbidity are shown as bold grey lines. Vertical bars represent the heatwaves in 2015. (DOCX 5663 kb)

## Data Availability

The dataset on emergency hospital admissions analyzed during the current study are available on request from the Federal Statistical Office Switzerland https://www.bfs.admin.ch/bfs/de/home/statistiken/gesundheit/erhebungen/ms.html

## Abbreviations

EHA: Emergency hospital admissions
ICD-10: International classification of disease, revision 10
Tmax: Daytime maximum temperature
Tmin: Nighttime minimum temperature
