# Supplementary material for: Impact of the warm summer 2015 on emergency hospital admissions in Switzerland
Source: Environ Health. 2019 Aug 15;18:66. doi: 10.1186/s12940-019-0507-1 (PMC6694501; doi:10.1186/s12940-019-0507-1)
Supplement: Supplementary file 1 — Additional tables and figures. Figure S1. Map of Switzerland showing the seven study regions and corresponding measurement station of temperature of the Swiss Monitoring Network. Figure S2. Daily number of emergency hospital admissions (EHA) (7 day moving average) in Switzerland by age group (0–14 years, 15–64 years, 65–74 years, ≥75 years) during the warm season between 2005 and 2015. External causes of morbidity are excluded except effects of heat and light (ICD10-code T67). Figure S3. Daily number of emergency hospital admissions (EHA) (7 day moving average) for selected diagnoses in Switzerland from 2005 to 2015. The reference years (2012–2015) for the 2015 excess morbidity are shown as bold grey lines. Vertical bars represent the heatwaves in 2015. (DOCX 5663 kb) [file 12940_2019_507_MOESM1_ESM.docx]

**Additional File 1**

**Impact of the warm summer 2015 on emergency hospital admissions in Switzerland**

Martina S. Ragettli^1,2^, Ana M. Vicedo-Cabrera^3^, Benjamin Flückiger^1,2^, Martin Röösli^1,2^

^1^ Swiss Tropical and Public Health Institute, Basel, Switzerland
^2^ University of Basel, Basel, Switzerland

^3^ Department of Public Health, Environments and Society, London School of Hygiene & Tropical Medicine, London, UK

Corresponding author:

Dr. Martina S. Ragettli

Swiss Tropical and Public Health Institute

Socinstrasse 57

P.O. Box

CH-4002 Basel

Martina.Ragettli@swisstph.ch

Tel: +41 61 284 87 29

**Figure S1.** Map of Switzerland showing the seven study regions and corresponding measurement station of temperature of the Swiss Monitoring Network.


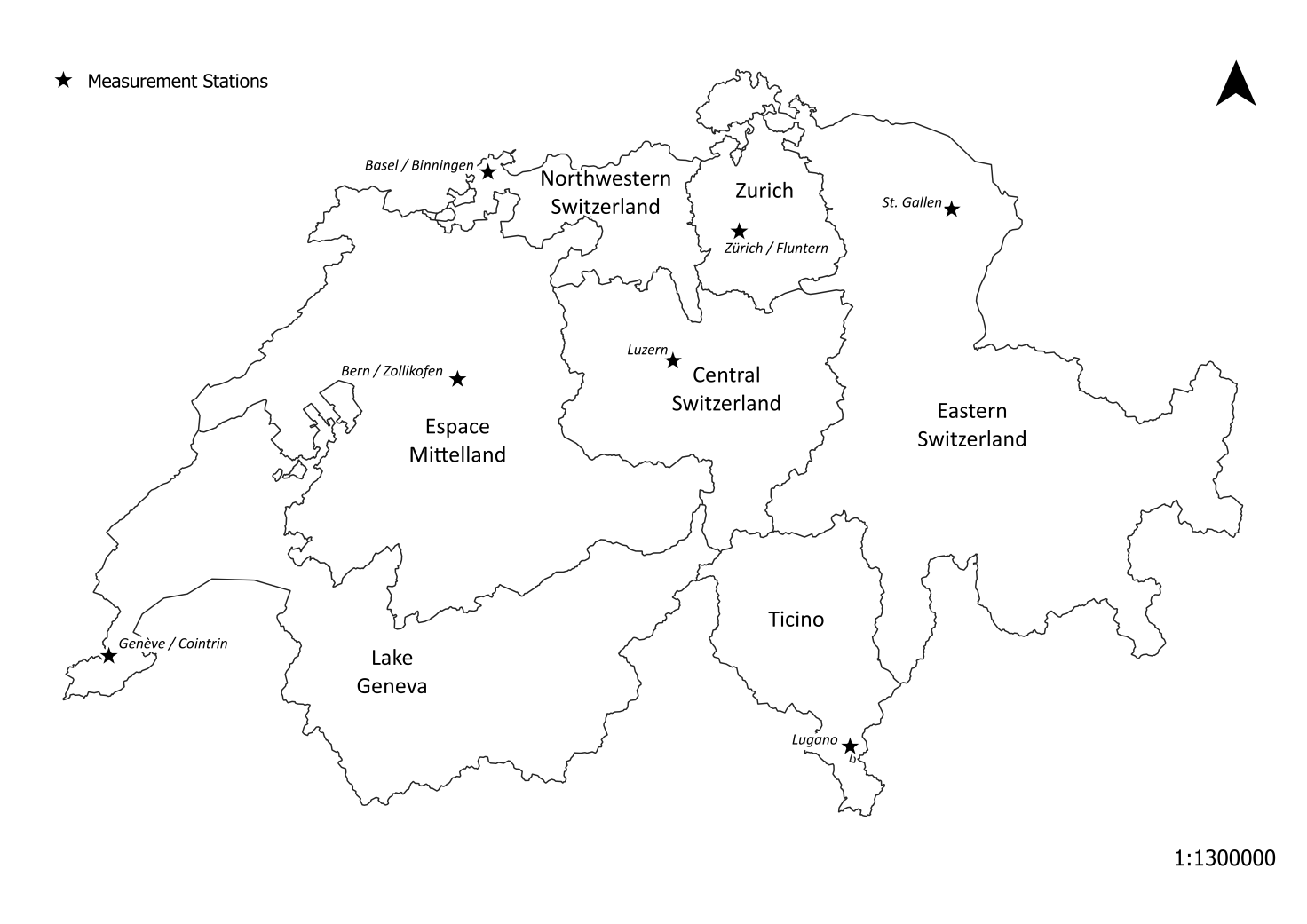


**Figure S2.** Daily number of emergency hospital admissions (EHA) (7 day moving average) in Switzerland by age group (0-14 years, 15-64 years, 65-74 years, ≥75years) during the warm season between 2005 and 2015. External causes of morbidity are excluded except effects of heat and light (ICD10-code T67).


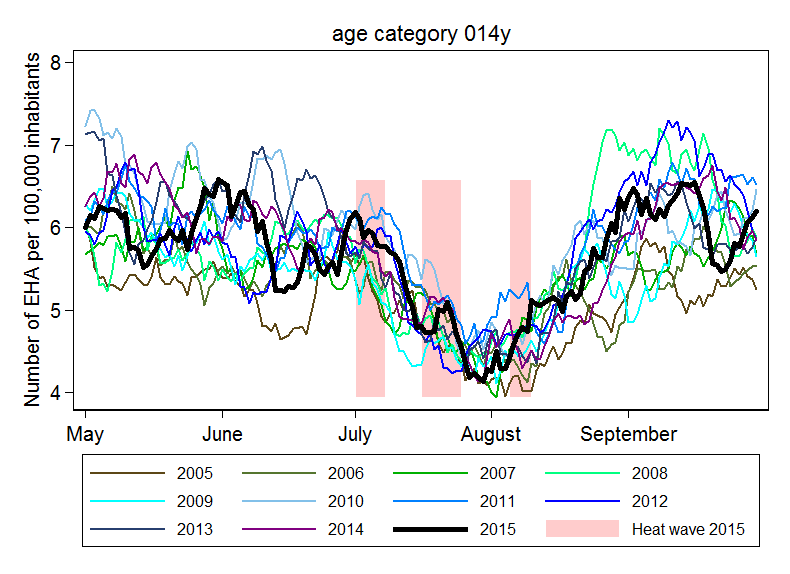

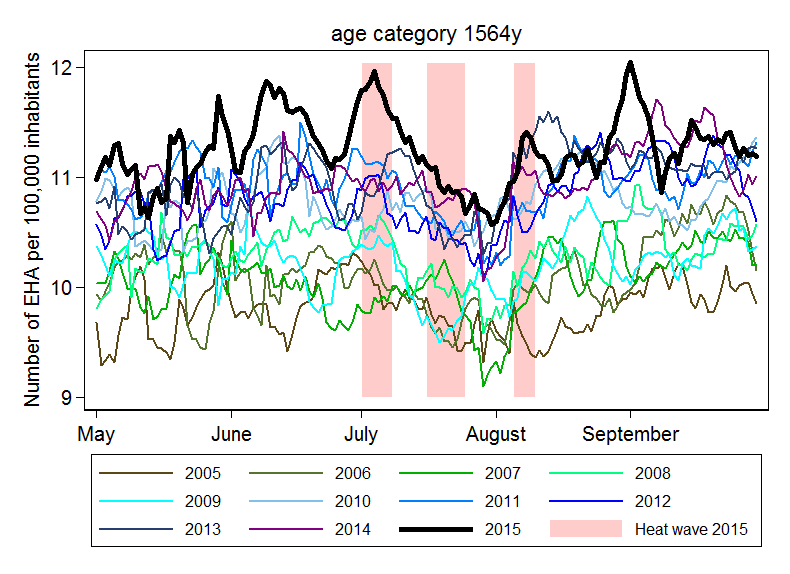


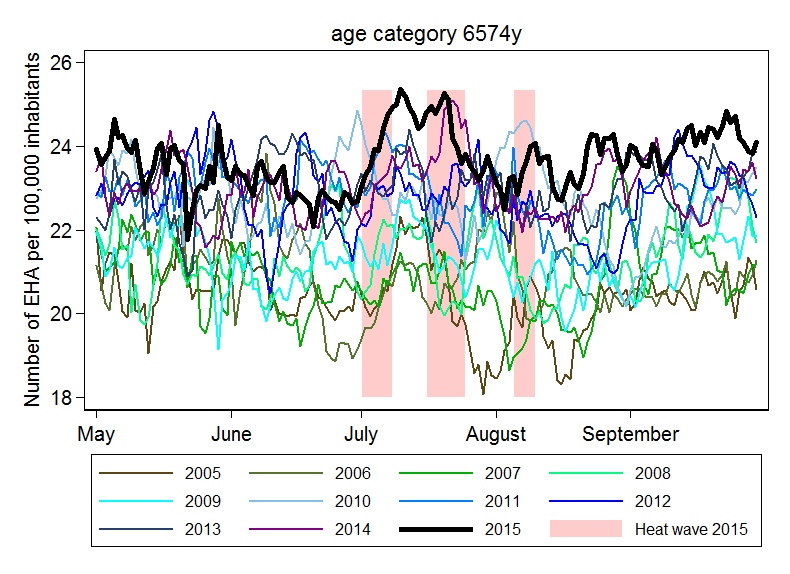

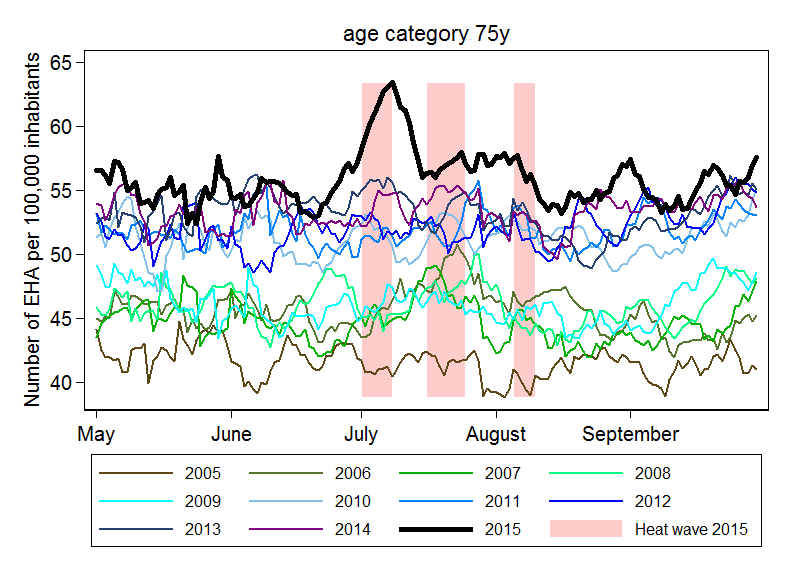


**Figure S3.** Daily number of emergency hospital admissions (EHA) (7 day moving average) for selected diagnoses in Switzerland from 2005 to 2015. The reference years (2012-2015) for the 2015 excess morbidity are shown as bold grey lines. Vertical bars represent the heatwaves in 2015.

**Figure S3. (cont.)**
